# Structural Characterization of a Therapeutic Anti-Methamphetamine Antibody Fragment: Oligomerization and Binding of Active Metabolites

**DOI:** 10.1371/journal.pone.0082690

**Published:** 2013-12-05

**Authors:** Eric C. Peterson, Reha Celikel, Kuppan Gokulan, Kottayil I. Varughese

**Affiliations:** 1 Department of Physiology and Biophysics, College of Medicine, University of Arkansas for Medical Sciences, Little Rock, Arkansas, United States of America; 2 Department of Pharmacology and Toxicology, College of Medicine, University of Arkansas for Medical Sciences, Little Rock, Arkansas, United States of America; University of Akron, United States of America

## Abstract

Vaccines and monoclonal antibodies (mAb) for treatment of (+)-methamphetamine (METH) abuse are in late stage preclinical and early clinical trial phases, respectively. These immunotherapies work as pharmacokinetic antagonists, sequestering METH and its metabolites away from sites of action in the brain and reduce the rewarding and toxic effects of the drug. A key aspect of these immunotherapy strategies is the understanding of the subtle molecular interactions important for generating antibodies with high affinity and specificity for METH. We previously determined crystal structures of a high affinity anti-METH therapeutic single chain antibody fragment (scFv6H4, K_D_ = 10 nM) in complex with METH and the (+) stereoisomer of 3,4-methylenedioxymethamphetamine (MDMA, or “ecstasy”). Here we report the crystal structure of scFv6H4 in homo-trimeric unbound (apo) form (2.60Å), as well as monomeric forms in complex with two active metabolites; (+)-amphetamine (AMP, 2.38Å) and (+)-4-hydroxy methamphetamine (p-OH-METH, 2.33Å). The apo structure forms a trimer in the crystal lattice and it results in the formation of an intermolecular composite beta-sheet with a three-fold symmetry. We were also able to structurally characterize the coordination of the His-tags with Ni^2+^. Two of the histidine residues of each C-terminal His-tag interact with Ni^2+^ in an octahedral geometry. In the apo state the CDR loops of scFv6H4 form an open conformation of the binding pocket. Upon ligand binding, the CDR loops adopt a closed formation, encasing the drug almost completely. The structural information reported here elucidates key molecular interactions important in anti-methamphetamine abuse immunotherapy.

## Introduction

 The abuse of methamphetamine (METH) is a significant societal problem in the United States and worldwide. Current pharmacological therapies for the treatment of the adverse health effects of stimulants such as METH relieve some organ-based symptoms caused by these harmful drugs. However, specific FDA-approved medications designed to treat the medical complications of METH abuse do not exist.

 Drug-specific immunotherapy is a promising approach to treating the adverse health effects of drug use for many important drugs of abuse, including nicotine [1], PCP [2], cocaine [3,4], methamphetamine [5–7] and others. By removing a drug from its sites of action or preventing it from reaching target sites, antibodies act as pharmacokinetic antagonists [8,9]. Unlike conventional receptor agonists or antagonists for treatment of drug abuse, antibodies have exquisite ligand or ligand class specificity and do not interfere with the actions of endogenous ligands or neurotransmitters, which can lead adverse effects. Moreover, since antibodies have extremely high affinities for their target ligand and do not cross the blood-brain barrier, they significantly lower drug concentrations in the central nervous system [10]. Thus, immunotherapies, and in this case anti METH immunotherapy, can provide broad neuroprotection to all sites of action in the central nervous system without causing any adverse effects in the brain. 

 Anti-METH monoclonal antibodies have the ability decrease brain concentrations of METH [11], reduce METH-induced behavioral effects such as locomotor activity [10], and have been shown to reduce the rate of self administration [5] in rat models of METH abuse. Since anti-METH antibodies do not rely on immune effector functions, such as antibody-dependent cell-mediated cytotoxicity, the intact IgG is not necessary for efficient function. A single chain antibody fragment (scFv6H4) was made from a high affinity antibody that is one-sixth the size of the parent IgG and was shown to rapidly decrease METH serum concentrations within a minute of intravenous administration in rats [12]. This shortened form offers potential advantages over the intact IgG form since only 1/3 of the protein dose is required for binding the same number of METH molecules as the IgG, and the sequence can be easily manipulated to create higher affinity mutants (unpublished work) and even conjugated to nanoparticles to customize *in vivo* properties [13]. 

**Figure 1:**
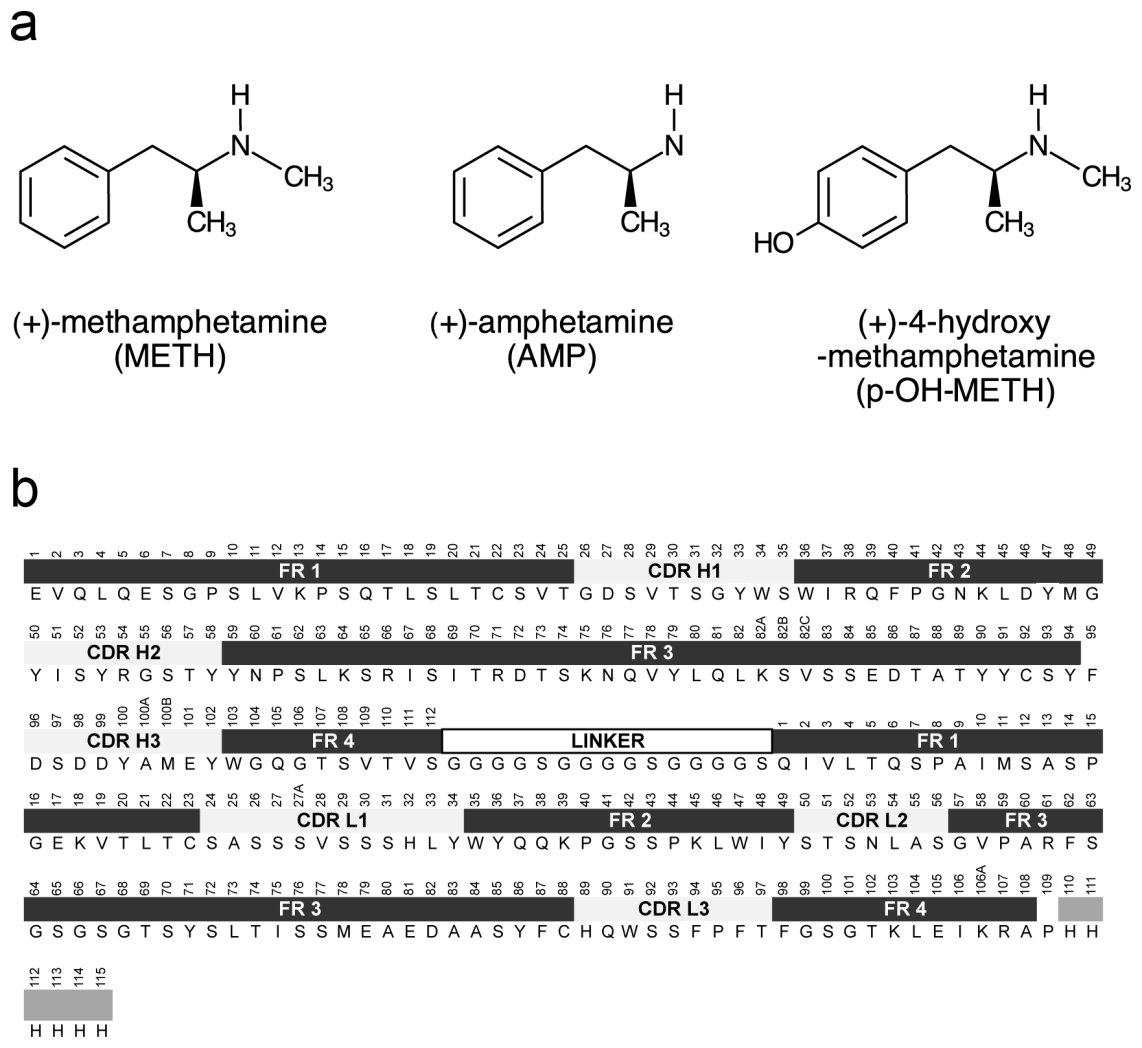
Structures of ligands and scFv6H4 sequence. (a) Structures of METH and metabolites AMP and p-OH-METH (b) Amino acid sequence of anti-METH scFv6H4. The variable heavy (H) and light (L) chains are numbered according to the Kabat numbering convention [29]. The location of the framework regions (FR), complementarity determining regions (CDRs), linker, and His-tag are indicated graphically above the amino acids. The antigen binding sites of antibodies are made up of three CDRs from the heavy chain and three from the light chain.

 A central aspect of designing immunotherapies for treating drug abuse, whether active vaccines, monoclonal antibodies, or antibody fragments, is the understanding of the mode of interaction between antibody and its target ligand. This structural understanding is important during development of the chemical haptens used to generate the antibodies [14] and understanding how the resulting antibodies bind the drug for further affinity improvements. This is especially important for a drug as small as METH (M.W. = 149.2), since the number of available molecular binding interactions are extremely limited. An additional challenge is discovering antibodies that will also bind to active METH metabolites (Figure 1), since a significant fraction of the original drug is converted to these metabolites in the body. Therefore, understanding the molecular interactions necessary to make an antibody specific to a drug class (e.g., METH-like stimulants), while showing limited affinity for endogenous ligands in the body is crucial. Toward elucidating these interactions, we previously solved the crystal structure of therapeutic anti-METH scFv6H4 in complex with METH and MDMA [15]. The present study was undertaken to extend our understanding of this therapeutic scFv by solving the structures of the empty site anti-METH scFv6H4, as well as in complex with two important active metabolites of METH, amphetamine and p-OH-METH (Figure 1a). The AMP complex and the p-OH-METH complex crystallized in the monomeric state similar to the METH complex, while the apo form crystallized as a homo-trimer stabilized by a nickel ion and a sulfate moiety. The main features of the three crystal structures are described below. The sequence of scFv6H4 is depicted in Figure 1b. 

## Results and Discussion

### Crystal structure ScFv6H4 in the free (apo) form

**Figure 2:**
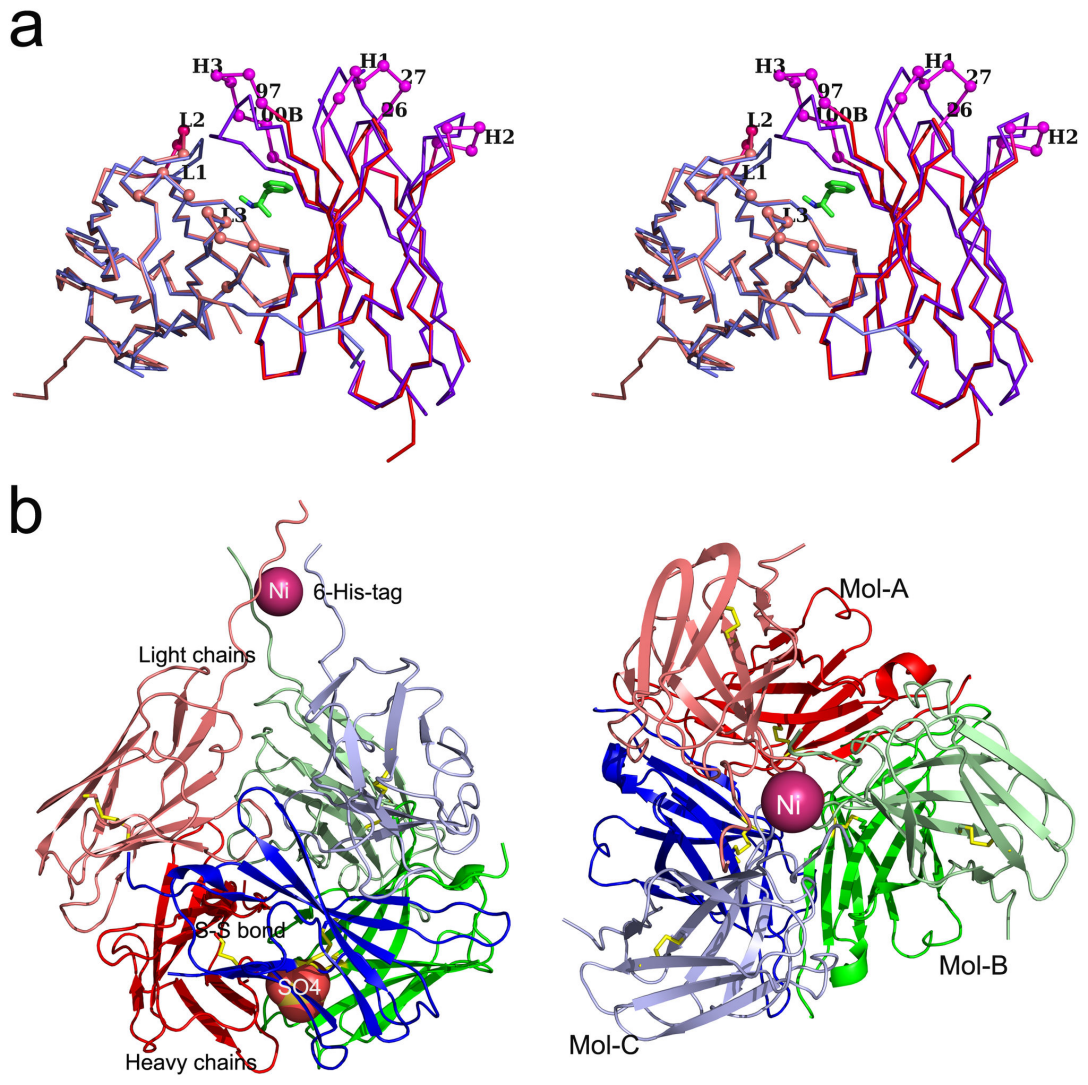
A comparison of the free form of scfv6H4 with the METH bound form. (a) A stereo-view depicting the superposition of the Cα atoms of the free form (pink) with the METH complex (purple). The largest deviations are all in the CDR loops. The METH is shown in green. The CDR H1, CDR H2 and CDR H3 are labeled as H1, H2 and H3. Likewise, CDR L1, CDR L2 and CDR L3 are labeled as L1, L2 and L3. Residues Gly H26, Asp H27, Ser H97 and Met H100B are labeled as 26, 27, 97 and 100B. The light chains are shown in lighter colors. (b) **Trimer formation**. Left panel: A lateral of a view cartoon representation of the trimer. The three molecules assemble around the 3-fold axis to give it a light bulb-like shape. The Ni^2+^ ion is shown as a sphere (in the stem region on the top) and the sulfate moiety (towards the bottom of the sphere) is shown as a CPK model. Right panel: A view from the top along the 3-fold axis. The three molecules are labeled as Mol-A (red), Mol-B (green) and Mol-C (blue). The light chains are shown in lighter colors.

The overall structure of scFv6H4 unbound to METH is similar to the previously reported structure of scFv6H4 in complex with METH [15] (Figure 2a). There are however significant differences in the conformation of the CDR loops and we will discuss them in detail in another section. The most conspicuous difference between the crystal structures of the apo form and the METH bound form is that the apo exists as a homo-trimer in the crystal lattice while the latter exists as a monomer. 

###  The homo-trimer of scFv6H4

The most intriguing aspect of the apo structure is the formation of the trimer in the crystal lattice (Figure 2b). The asymmetric unit contains only one molecule, but it uses the lattice symmetry to form the homo-trimer. For the sake of description, we will designate the three molecules as Mol-A, Mol-B and Mol-C. The scFv6H4 sequence contains a His-tag at the C-terminal which interacts with the Ni^2+^ ion present in the crystal lattice. The trimer is shaped like a light bulb with a stem-like upper region and a spherical region. The stem-like region consists of the C-terminal residues including the His-tag residues. The organization of the spherical region and the stem region are described below. 

###  The architecture of the spherical region

ScFv6H4 is comprised of two main domains – the variable heavy chain domain (V_H_) and the variable light chain domain (V_L_) both with the characteristic immunoglobulin fold. This fold consists of two β-sheets sandwiched to form a hydrophobic core where the two sheets are bridged by a disulfide bond. The β-sheets are made up of anti-parallel β-strands. One sheet is comprised of 5 strands and the other of 3 strands. When the heavy and light chains associate the larger sheets of the two domains form the interior and the smaller sheets form the exterior. 

 The six domains of Mol-A, Mol-B and Mol-C arrange to form a sphere (Figure 2b). The V_L_ domains form the upper hemisphere and the V_H_ domains form the lower hemisphere. The formation of the trimer involves extensive surface contacts and buries 22.7% of the surface area of each molecule. In fact, this contact area is significantly larger than the area buried when the light chain and the heavy chain associate to form a functional unit (around 10%). The lower hemisphere is tightly packed while the upper hemisphere is loosely packed. In the spherical portion, most of the significant adhesive interactions are mediated through the heavy chain, involving the formation of a composite β-sheet and adhesive interactions through a sulfate moiety. The interactions at the V_L_ neck (stem) are mediated through the Ni^2+^ ion. 

###  Trimer forming Interactions

In addition to the interactions mediated through the Ni^2+^ ion, there are other prominent interactions contributing to the formation and stabilization of the trimer, such as the fusion of three β-sheets into one composite sheet and the interaction through a sulfate group as described below. 

###  The composite β-sheet

**Figure 3:**
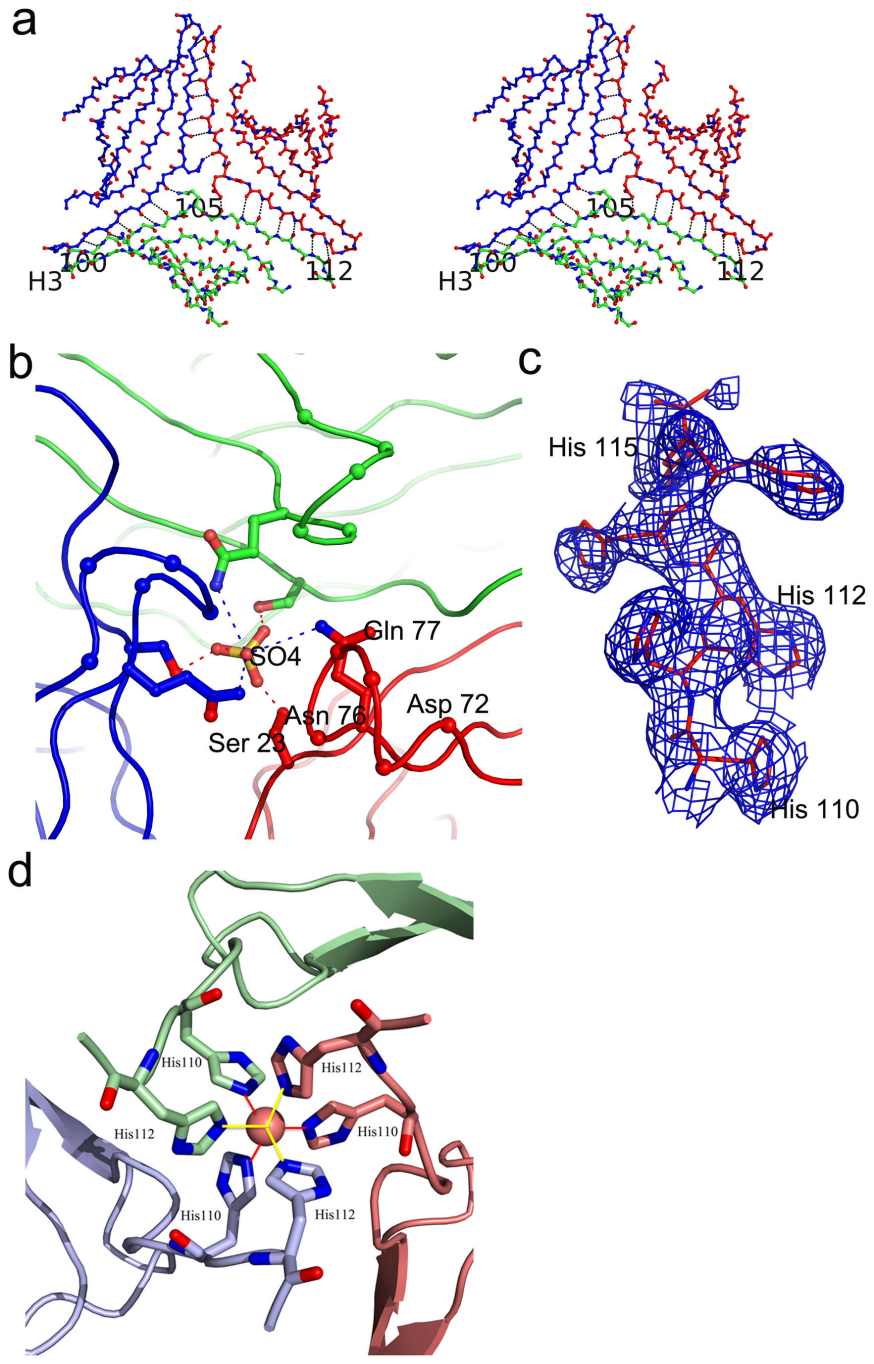
Trimer forming interactions. (a) A stereo view of the composite β-sheet: One of the β-sheets of the V_H_ consists of five β-strands and it joins together with identical sheets from the symmetry related molecules to form a composite β-sheet with a 3-fold symmetry. The outer strand (Tyr H100 to Ser H112) is the longest, with a significant bend which separates it into two segments. The two segments interact with separate molecules joining the three sheets together. The residues Tyr H100, Gln H105 and Ser H112 are labeled as 100, 105 and 112 respectively. The location of the CDR H3 loop is labeled as H3. (b) Interactions of the sulfate moiety: The sulfur atom and one of the oxygen atoms sits on the crystallographic 3-fold axis. The oxygen atom on the three fold interacts with three Gln residues (Gln H77 of the three V_H_) related by symmetry. The other oxygen atoms interact with Ser residues (Ser H23 of the three V_H_). (c) A view of the electron density of the His-tag residues. This diagram depicts the 2Fo-Fc electron density map of the His-tag residues. The contours are drawn at 1.2 σ level. (d) Ni-coordination: The Ni ^2+^ ion sits on the crystallographic 3-fold symmetry axis coordinating with 6 Histidine residues in octahedral geometry. Each of the symmetry related chains contributes two histidine residues for coordination. The bound nickel is shown as a sphere and histidine residues are represented as stick models. The three symmetry related molecules are shown in green, cyan and pink.

Here the 5-stranded β-sheets from the three molecules come together to form a composite β-sheet with a 3-fold symmetry (Figure 3a). The sheets are linked through an extensive network of hydrogen bonds. Relevant to our discussion is the conformation of the CDR H3 loop which links the two adjacent β-strands on the outer edge of the sheet. In the METH-bound form, these strands extend from residue Thr H87 to Asp H96 and from Tyr H102 to Ser H112, respectively. In the apo structure, the CDR H3 loop transforms its conformation and one strand is elongated by two peptide units (Thr H87-Asp H98) and the other by four peptide units (Tyr H100-Ser H112) to maximize the intermolecular interactions. During the molecular assembly, the outermost β-strand (Tyr H100-Ser H112) serves as the connecting strand with the other sheets. The strand is bent into two segments enabling it to interact with two strands. The interactions of both segments lead to the formation of anti-parallel β-strands. The composite β-sheet has an apparent “doughnut hole” in the middle considering only the backbone peptide atoms. In reality, however, this hole is filled by the Gln H105 residues from the three molecules. The side chain nitrogen atom NE2 forms a hydrogen bond with the carbonyl of Gly H106 from an adjacent molecule further stabilizing the formation of the composite β-sheet (Figure 3a). 

###  Sulfate Interactions

The sulfate moiety is located below the composite β-sheet and is involved in adhesive interactions at two levels with framework regions 1 and 3 of scFv6H4. It occupies a symmetric position with the sulfur atom and one of the oxygen atoms (O4) sitting on the crystallographic 3-fold axis. On one level, the sulfate oxygen atoms O1, O2, and O3 interact with the side chain of Ser H23 (2.5Å) from the three molecules (Figure 3b). 

 In the lower level, The O4 atom of the sulfate interacts with the residue Gln H77 residues from the three molecules (O…N distance 3.2 Å, Figure 3b). In addition to this adhesive link, this peptide region has other interactions which link the trimer together. The residues Asp H72-Gln H77 form a β-bend and these residues from the three molecules come close together around the 3-fold axis to generate more hydrogen bonds. The side chain of Lys H75 interacts with two carbonyl atoms in the next molecule. In addition, the side chain from Gln H77 interacts with the side chain of Asn H76 from an adjacent molecule. 

### Nickel coordination with the His-tag residues

The nickel ion is situated at the top in the stem region (Figure 2b). One of the most fascinating aspects of this structure is that all the His-tag residues are visible in the electron density map (Figure 3c) and the Ni^2+^ atom interacts with the His-tag residues. The Ni^2+^ atom sits on the crystallographic 3-fold axis and two histidine residues (His 110 and His 112) from each of the three molecules related by the 3-fold symmetry coordinate with Ni^2+^ to form an octahedral coordination (Figure 3d). The Ni^2+^-nitrogen distances of coordination are around 2.2 Å. These interactions play a role in stabilizing the trimer if not initiating its formation. There are many reports of metal ion binding mediating or stabilizing molecular adhesion such as α-subunit of integrin CR3 and RNase A [16,17]. It can also play a role in crystallization [18]. In this context, a methodology for promoting crystallization through metal binding has also been proposed [19]. This technique is to engineer metal binding sites such as Cu^2+^, Zn^2+^ and Ni^2+^ to protein surfaces in order to induce symmetric aggregation into crystal lattices. In the present structure of the apo form, it is evident that the symmetry of the crystal lattice is correlated with the nickel coordination of the histidine residues.

###  Conformational changes in the apo structure

**Figure 4:**
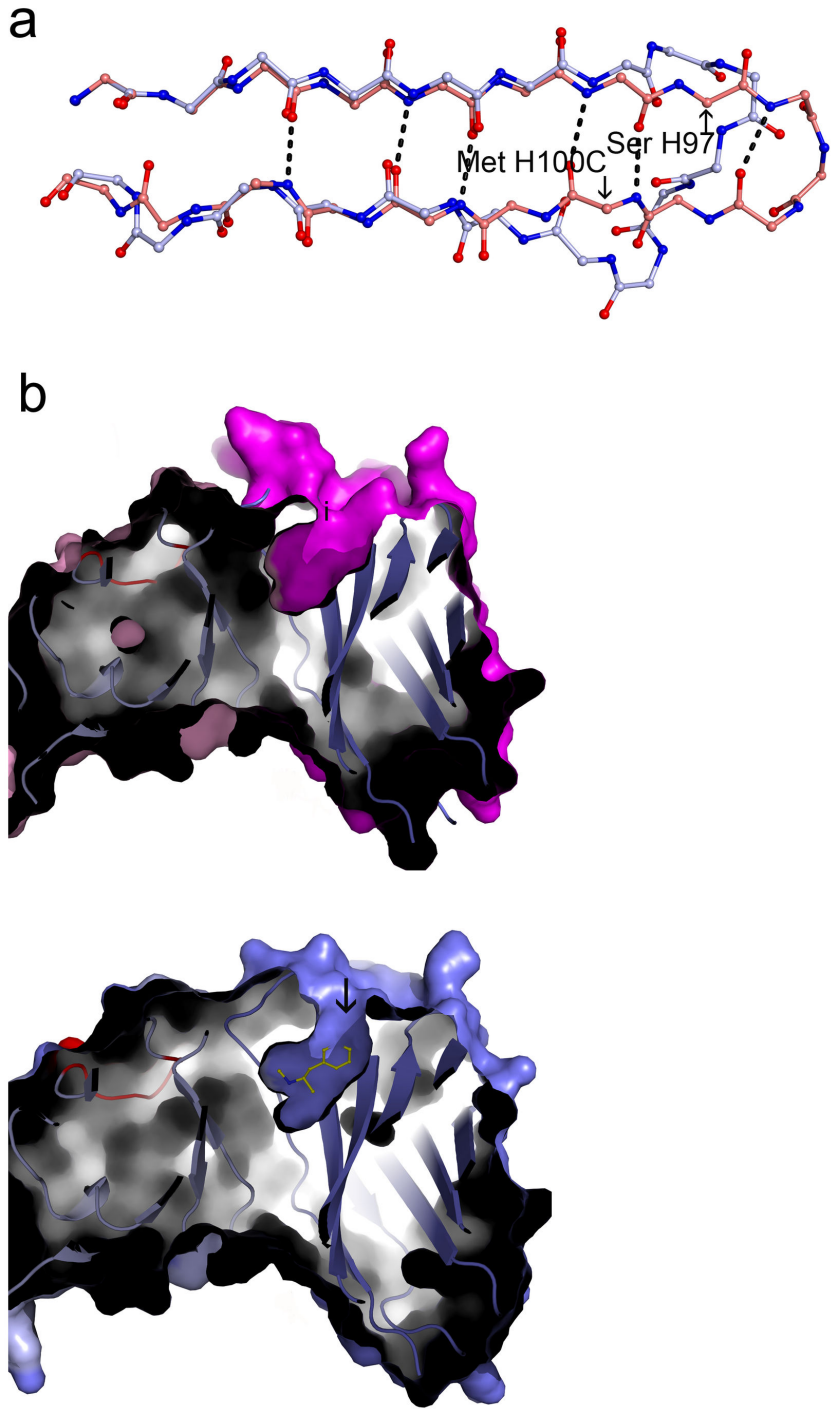
Conformational changes upon trimer formation. (a) CDR H3 loop: A superposition of the Cα atoms of residues Thr H89-Thr H107 in the apo structure (magenta) with the METH bound structure (yellow). Six residues belonging to the CDR H3 loop (Ser H97 to Met H100B) show significant displacements. In the apo crystal, the loop residues take up extended conformations elongating the two anti-parallel β-strands to have a sharp turn connection in the place of a long loop. In the METH-bound structure, the CDR H3 region assumes a more spread out conformation to create the environment for METH binding. (b) The shapes of the binding pockets in the free and the antigen bound states: The left panel (apo form) and the right (METH complex) show identical cross sections. The entrance of the binding pocket is broader in apo structure, compared to the METH complex. **Color code**: Left panel: heavy chain -magenta and light chain -light pink. Right panel: heavy chain- blue light chain-light blue.

As seen in Figure 2a, the overall structure of the apo form is similar to the METH bound form, but there are significant changes in the CDR loops, most significantly in the CDR H3 loop. These large shifts are due to its conformational transition into a β-hairpin in the apo structure. Six residues in this loop (Ser H97-Met H100B) show significant displacement with Tyr H100C showing the largest deviation (6.5 Å) in the Cα positions. In the METH bound structure, the connection between the two β-strands is through a large loop (CDR H3), while in the apo structure, the strands elongate to form a β-hairpin with a sharp turn (Figure 4a). This transformation has significant role in stabilizing the trimeric state as we have seen before. A few residues in the CDR H1 loop also show large displacements with both Gly H26 and Asp H27 Cα positions shifting around 6Å. There are displacements in the CDR H2 loop also, but they are smaller and are around only 1Å. In the light chain, CDR L1 and CDR L3 loops do not deviate much, but CDR L2 loop shows deviations around 2.8 Å (residues Ala L55, Ser L56, Gly L57, Val L59 and Pro L59). These movements result in a more open pocket in the apo form compared with the METH bound form (Figure 4b). The electron density map showed the presence of one ordered water molecule in the binding pocket, compared with two in the METH-bound structure.

###  His-tags in crystallography

In the production of recombinant proteins, His–tags are widely used to expedite protein purification procedures and to achieve high levels of purity. Initially there were concerns that the presence of a histidine tag would influence the structure of the protein, but it has been shown that these affinity tags do not affect the 3-D structure of protein molecules [20]. It is however rather rare to be able to locate histidine tag residues in crystal structures as they are usually not well defined in electron density maps. It has been pointed out that in all of the reported crystal structures containing His-tag residues, less than 6% could locate four or more consecutive His residues and only in only 2.5% of the cases five or more His residues could be observed [20]. Despite the widespread use of histidine tags, as far as we know the structural characterization of histidine coordination with a Ni^2+^-ion has not been reported so far. 

 We did not add Ni^2+^ deliberately into the crystallization solutions, thus we assume that these ions were bound by the His-tag during column purification and retained throughout the protein concentration and buffer exchange steps. We used NiSO4 to “charge” the IMAC column initially and regenerate the column between purifications. Since no NiSO4 containing buffers were used in any downstream processes of protein purification, we assume that the Ni^2+^ was carried forward in the purification process even after extensive buffer exchange. 

###  Oligomer structure relevance to previous preclinical studies

Although homo-oligomerization of scFvs into dimers, trimers and sometimes tetramers is well known [21,22], the common mechanism for formation is an association of a variable heavy region of one chain with the variable light region of another, unlike the associations that we see in the scFv6H4 apo structure. The formation of an oligomeric state is often dependent upon the length of the linker between domains, with shorter linkers favoring higher order oligomers [23]. A linker of 15 amino acids, as we have engineered into scFv6H4 should promote the formation of mostly monomeric forms. Indeed, the purified and buffer exchanged scFv6H4 is a mixture of roughly 65% monomer, 25% dimer and 10% trimer [12]. Dolezal et. al [22] observed that the anti-neuraminidase scFv exists as trimers and tetramers, however the model they proposed involves swapping of domains in a cyclic manner either to form trimers or tetramers in contrast to the assembly of intact scFv6H4 molecules observed in the present case. Interestingly, we have observed spontaneous conversion of some monomer to multimeric forms *in vivo* also, but we could not determine a mechanism [12]. 

###  Structure of scFv6H4 in complex with AMP

**Figure 5:**
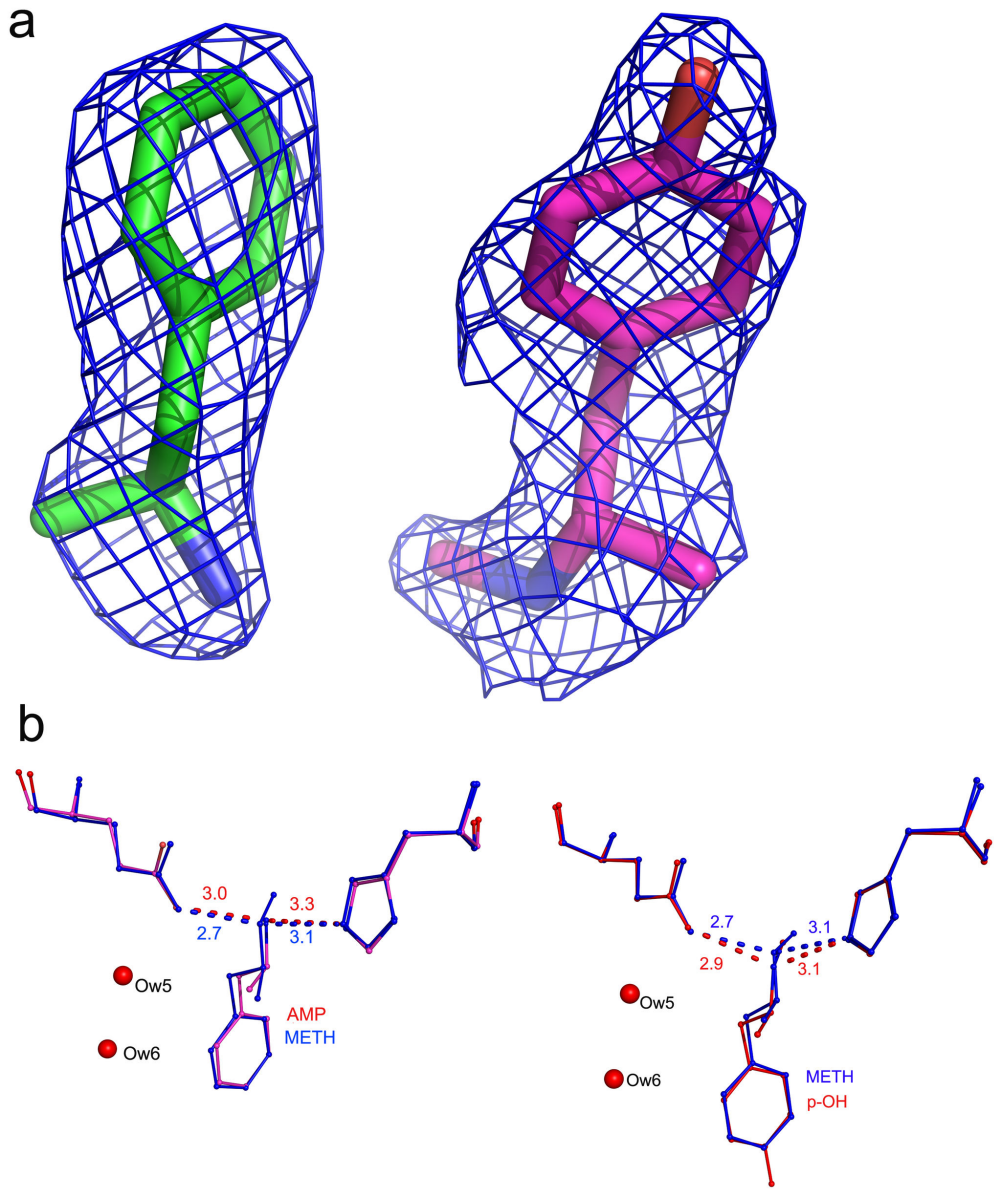
AMP and p-OH-METH binding. (a) Electron density of the ligands: Left panel: A view showing the electron density (2Fo-Fc) of AMP. Right panel: A 2Fo-Fc map of p-OH-METH. The contours are drawn at 1.2 σ level. (b) A comparison of the binding modes of the two metabolites with METH. Left panel: A superposition of AMP (pink) with METH (blue). The antibody binds both AMP and METH in a similar manner, but there is a shift in the position of the nitrogen atom which forms hydrogen bonds with Glu H101 of the V_H_ chain and His H89 of the V_L_ chain. The hydrogen bond distances indicate that in the AMP structure they are much weaker. Right panel: p-OH-METH: The geometry of the binding pocket is the same in the METH structure and the hydrogen bonds are of comparable strength as in the METH structure.

AMP is the demethylated form of METH, but with substantially lower binding affinity to scFv6H4 (K_D_ = 20.7 μM). Despite the lower affinity, clear density was observed in the electron density map (Figure 5a). AMP binds scFv6H4 in a very similar manner as METH, but a comparison of the two structures reveals that the cationic nitrogen in the AMP structure is shifted by about 0.78 Å from its position in the METH complex (Figure 5b). Consequently, the interactions of the nitrogen are significantly weaker. In the METH complex, the nitrogen has a strong salt bridge/hydrogen bond interaction with Glu H101 (N…O distance of 2.7Å). There is a second hydrogen bond with His L89 with the distance of 3.1 Å. In the AMP structure these two distances are 3.2Å and 4.0Å, respectively making them much weaker (Figure 5b). We attribute the reduction in binding affinity to looser packing and weakened hydrogen bonding interaction. Elucidation of this weaker interaction with AMP explains earlier *in vitro* and *in vivo* observations with scFv6H4 and the parent monoclonal antibody anti-METH mAb6H4 [10,12,13]. 

 As in the METH structure, the binding cavity in the AMP structure also contains two water molecules. Using *in silico* modeling, others have proposed that more water molecules actually occupy the binding pocket in the METH and AMP complex, but these water molecules evaporated during the crystallization process [24,25]. However, we do not consider the additional water molecules as plausible and we have not seen evidence of additional waters in the three scFv6H4:drug structures that we have solved. Interestingly, though the size of AMP is smaller than METH, there is no evidence for any additional water molecules in the cavity. 

###  Structure of scFv6H4 in complex with p-OH-METH

p-OH-METH is an active metabolite that is converted in the liver. Up to 15% of the METH dose ingested is converted to p-OH-METH [26], thus a pharmacokinetic antagonist that is able to bind p-OH-METH efficiently could have increased efficacy. Although we have not determined the affinity of scFv6H4 for p-OH-METH, we expect it to be very close to the affinity of the parent mAb6H4, which is 18.7 nM. The structure was analyzed with 2.33Å diffraction data. ScFv6H4 binds p-OH-METH tightly and Figure 5a depicts the electron density antibody of the ligand. The mode of p-OH-METH binding is the same as in the METH complex of ScFv6H4. Figure 5b shows a superposition of p-OH-METH and METH in the two crystal structures. The hydrogen bonds of the cationic nitrogen remain intact. The binding pocket retains the two water molecules and maintains all the hydrophobic interactions. As the hydroxyl group of p-OH-METH points outwards, it does not in any way interfere with any of its interactions with binding pocket and therefore the binding is equally strong as for METH. 

## Conclusion

 Here we report crystallographic structures of a therapeutic anti-METH antibody in the empty site (apo) conformation and in complex with AMP and p-OH-METH. Although the antibody was raised using a METH-like hapten, it binds to its metabolites AMP and p-OH-METH also. AMP is a smaller molecule lacking a methyl group and p-OH-METH possesses an extra hydroxyl group compared to METH. The crystal structures provide lucid explanation for the poor binding of AMP and the tight binding of p-OH-METH. 

 Both hydrophobic and hydrophilic forces are in play to keep the ligand bound in the pocket. In all three cases, hydrophobic interactions are invariant. Between METH and AMP, the differences are mainly in hydrophilic interactions – the two hydrogen bonds of the cationic nitrogen atom. In the case of AMP binding, the two hydrogen bonds are significantly weaker accounting for the lower affinity. In the case of p-OH-METH, the hydroxyl group points to the solvent and does not create any steric hindrances in the binding pocket. Therefore all the binding interactions are preserved as in METH and explaining the tight binding of p-OH-METH. 

 In the apo structure, the binding pocket assumes an open conformation. When scFv6H4 binds a ligand, the CDR loops undergo conformational changes and move towards the ligand to interact with it. The apo form crystallized with a Ni^2+^ ion sitting on a crystallographic three fold axis and the His-tag residues from three symmetry molecules cluster around it and form an octahedral coordination. The most intriguing aspect of the apo structure is that it forms a trimer in bringing three β-sheets together to form a composite β-sheet with a 3-fold symmetry. 

## Materials and Methods

###  Chemicals and Drugs

All chemicals were purchased from Sigma-Aldrich (St. Louis, MO) unless otherwise noted. Enzymes and Escherichia coli strains were purchased from Invitrogen (Carlsbad, CA).

###  Cloning, expression and purification of scFv6H4

Cloning was carried out as described earlier [12,15]. Briefly, scFv6H4 was cloned by connecting the variable heavy (VH) and variable light (VL) chains with cDNA encoding a 15 amino acid linker in a VH-Linker-VL orientation. A His-tag was included at the C-terminus for ease of purification. The coding sequence cassette was ligated into plasmid pPICZ-alpha (Invitrogen, Carlsbad, CA) to form an expression plasmid with a secretory signal and a methanol-inducible promoter. Once the sequence was confirmed, the plasmids were used to transform yeast strain *Pichia pastoris* for expression. ScFv6H4 was purified after expression by immobilized metal affinity chromatography on a His-Trap FF column (GE Healthcare, Piscataway, NJ) that was activated with NiSO_4_. After purification, the protein was concentrated and the buffer was exchanged by centrifugal concentration.

###  Crystallization

Crystals of the scFv6H4:p-OH-METH complex and the scFv6H4:AMP complex were grown using hanging drop vapor diffusion technique as follows. Scfv6H4 stocks were concentrated to 12 mg/ml in a solution of 5 mM HEPES, 150 mM sodium chloride and 5 mM octyl β-D- glucopyranoside detergent at pH 8.3. AMP or p-OH-METH was added to the protein solution to a final concentration of 5 mM to obtain a protein to ligand ratio of 1:5. The reservoir solution contained 1.15M sodium citrate and 0.28M imidazole-malate (at pH 8 for METH complex and pH 7.8 for p-OH-METH. The hanging drops were made by combining 1 μl of the protein stock with 1 μl reservoir solution on 22 mm microscope cover slips. The crystallization tissue culture plates were kept at 14° C in an incubator. 

 The crystals of the apo form were grown in a similar fashion also using hanging drop methods. The scFv6H4 was concentrated to 14 mg/ml in 5 mM HEPES, 100mM NaCl, 1 mM NaN_3_ at pH 8.0 and reservoir solution contained 1.6 M ammonium sulfate, and 240 mM imidazole malate at pH 8.0. The hanging drops were prepared by mixing 1 μl of the protein stock with 1 μl reservoir solution as before. All crystals grew in about 3 weeks. 

###  Data Collection and Processing

The data sets for all three crystals were collected at the Stanford Synchrotron Radiation Lightsource (SSRL), Palo Alto, California. The summary of data collection statistics of both crystals are listed in Table 1.

**Table 1:** Data Collection and Refinement Statistics.

**Crystal**	**scFv6H4 apo form**	**scFv6H4:AMP**	**scFv6H4: p-OH-METH**
Space group	P2_1_3	P2_1_	P2_1_
Cell dimensions: a,b,c(Å); α, β, γ (°)	88.34, 88.34, 88.34; 90.00, 90.00, 90.00	34.55, 65.23, 48.59; 90.00, 98.70, 90.00	34.28, 65.30, 48.57; 90.00, 98.36, 90.00
Resolution (Å)	2.80	2.38	2.33
Rmerge	8.2% (50)	9.3% (33)	5.3 (12.6)
Average *I*/ σ*I*	45.8 (6.2)	24.9 (4.3)	32.4 (10.6)
Completeness (%)	99.8 (99.7)	93.3 (92.2)	93.7 (92.8)
Wavelength	0.97946	0.97946	0.97946
Number of Images	135	321	291
Total Number of observations	77,178	34836	25666
Unique reflections	7243	7947	8577
Redundancy	10.7	4.4	3.0
**Refinement Statistics:**			
Protein atoms	1726	1778	1777
Ligand atoms	0	10	12
Water molecules	9	55	71
Rfactor/Rfree	20.77/26.98	21.85/21.29	16.38/23.75
Rms bond lengths/ angles	0.013 Å1.59 Å	0.018 Å /1.86 Å	0.014 Å /1.59 Å
**Ramachandran Statistics**			
Most favored	84.4%	92.98 %	95.18%
Additionally allowed	10.9%	4.82%	3.51%
Disallowed	5.5%	2.19%	1.32%
Luzzati Error	0.30	0.26	0.275
**Average B values (Å^2^)**			
Protein	34.15	28.64	28.8
Ligand	none	36.87	20.96
Solvent molecules	49.94	30.97	29.873

The values in the parentheses refer to the highest resolution shell. The diffraction data were collected at 100°K on beam line 9-1 and 9-2 at Stanford Synchrotron Radiation Laboratory, Palo Alto, California. The data were reduced using the software package HKL2000 [30].

###  Structure Determination of scFv6H4 free form

The apo form crystallized in the cubic space group P2_1_3, different from the crystals of the METH complex, which belonged to monoclinic space group P2_1_. The structure was solved employing molecular replacement techniques with the known structure of the scFv6H4:METH complex as the starting model (PDB # 3GKZ). Using 15-4Å data, the cross rotation searches and subsequently translation function searches were carried out with the CNS suite of programs [27]. The structure was refined using simulated annealing and restrained positional refinement. While the overall structure was very similar to that of the METH complex, most of the CDR loops had significantly different orientations and they were rebuilt using the 2Fo-Fc maps using Win coot. The final refinements were carried out using Refmac5 in CCP4 package [28]. 

###  Structure determination of scFv6H4:OH-METH and scFv6H4:AMP

Both of these binary complexes crystallized in the same P2_1_ space group as the METH complex [15]. Therefore using the METH structure as the starting model, following rigid body refinements electron density maps were examined and appropriate adjustments were made to the model. The models were further refined initially using CNS suite of programs [27] and subsequently using Refmac5 in the CCP4 package, and final model was inspected using Wincoot.

### Accession numbers

The atomic coordinates and structure factors for scFv6H4:p-OH-METH (Research Collaboratory for Structural Bioinformatics Protein Databank = PDB # 4LAS and scFv6H4:AMP (Research Collaboratory for Structural Bioinformatics Protein Databank = PDB # 4LAR and scFv6H4 free form (Research Collaboratory for Structural Bioinformatics Protein Databank = PDB # 4LAQ have been deposited in the Research Collaboratory for Structural Bioinformatics Protein Databank, Rutgers University, New Brunswick, NJ (http://www.rcsb.org).
