# Correction: Structural Characterization of a Therapeutic Anti-Methamphetamine Antibody Fragment: Oligomerization and Binding of Active Metabolites

**DOI:** 10.1371/annotation/f92e9067-daee-45af-b5da-5779273225ed

**Published:** 2014-01-02

**Authors:** Eric C. Peterson, Reha Celikel, Kuppan Gokulan, Kottayil I. Varughese

While preparing this article for publication, Figure 2a was re-sized to a form unsuitable for stereo glasses. Please view Figure 2a in its original size here: http://plosone.org/corrections/pone.0082690.g002a.cn.tif

The legend for Figure 4b refers to left and right panels rather than top and bottom panels. The correct legend for Figure 4b should read:

The shapes of the binding pockets in the free and the antigen bound states: The top panel (apo form) and the bottom (METH complex) show identical cross sections. The entrance of the binding pocket is broader in apo structure, compared to the METH complex. Color code: Top panel: heavy chain -magenta and light chain -light pink. Bottom panel: heavy chain- blue light chain-light blue. 

